# Feasibility of HER2-Targeted Therapy in Advanced Biliary Tract Cancer: A Prospective Pilot Study of Trastuzumab Biosimilar in Combination with Gemcitabine Plus Cisplatin

**DOI:** 10.3390/cancers13020161

**Published:** 2021-01-06

**Authors:** Hyehyun Jeong, Jae Ho Jeong, Kyu-Pyo Kim, Sang Soo Lee, Dong Wook Oh, Do Hyun Park, Tae Jun Song, Yangsoon Park, Seung-Mo Hong, Baek-Yeol Ryoo, Changhoon Yoo

**Affiliations:** 1 Asan Medical Center, Department of Oncology, University of Ulsan College of Medicine, Seoul 05505, Korea; 0315jhh@gmail.com (H.J.); jaeho.jeong@amc.seoul.kr (J.H.J.); kkp1122@amc.seoul.kr (K.-P.K.); ryooby@amc.seoul.kr (B.-Y.R.); 2 Asan Medical Center, Department of Gastroenterology, University of Ulsan College of Medicine, Seoul 05505, Korea; ssleedr@amc.seoul.kr (S.S.L.); wooki219@naver.com (D.W.O.); dhpark@amc.seoul.kr (D.H.P.); medi01@naver.com (T.J.S.); 3 Asan Medical Center, Department of Pathology, University of Ulsan College of Medicine, Seoul 05505, Korea; ysp@amc.seoul.kr (Y.P.); smhong28@gmail.com (S.-M.H.)

**Keywords:** biliary tract cancer, HER2, trastuzumab-pkrb, targeted therapy

## Abstract

**Simple Summary:**

Unresectable or metastatic biliary tract cancer (BTC) has a poor prognosis with the standard gemcitabine and cisplatin (GemCis) regimen. Given the considerable incidence of HER2-overexpressing tumours (e.g., >10% of gallbladder cancer), HER2 is a potential therapeutic target in advanced BTC. In this prospective study, 7 out of 41 (17.1%) patients had HER2-positive tumours, and 4 patients (9.8%) subsequently proceeded to receive HER2-targeted therapy. The combination of trastuzumab-pkrb, an anti-HER2 monoclonal antibody, and GemCis resulted in high overall response (50%) and disease control (100%) rates in HER2-positive advanced BTC patients without new safety issues. This is the first prospective study that suggested the feasibility of HER2-targeted combination chemotherapy in advanced BTC patients. Future prospective randomised trials using HER2-targeted agents are warranted.

**Abstract:**

The prognosis of advanced biliary tract cancer (BTC) is poor with the standard gemcitabine and cisplatin (GemCis) regimen. Given that the rates of human epidermal growth factor receptor 2 (HER2) positivity in BTC reaches around 15%, HER2-targeted therapy needs further investigation. This study aims to evaluate the preliminary efficacy/safety of first-line trastuzumab-pkrb plus GemCis in patients with advanced BTC. Patients with unresectable/metastatic HER2-positive BTC received trastuzumab-pkrb (on day 1 of each cycle, 8 mg/kg for the first cycle and 6 mg/kg for subsequent cycles), gemcitabine (1000 mg/m^2^ on day 1 and 8) and cisplatin (25 mg/m^2^ on day 1 and 8) every 3 weeks. Of the 41 patients screened, 7 had HER2-positive tumours and 4 were enrolled. The median age was 72.5 years (one male). Primary tumour locations included extrahepatic (N = 2) and intrahepatic (N = 1) bile ducts, and gallbladder (N = 1). Best overall response was a partial response in two patients and stable disease in two patients. Median progression-free survival (PFS) was 6.1 months and median overall survival (OS) was not reached. The most common grade 3 adverse event was neutropenia (75%), but febrile neutropenia did not occur. No patient discontinued treatment due to adverse events. Trastuzumab-pkrb with GemCis showed promising preliminary feasibility in patients with HER2-positive advanced BTC.

## 1. Introduction

Biliary tract cancer (BTC) is a group of malignancies arising from the epithelium of the biliary tree, including gallbladder, extrahepatic and intrahepatic cholangiocarcinoma. It is a rare and heterogeneous disease group. In the US, about 12,000 new cases of BTC are reported annually [1], although the incidence of BTC varies according to the geographic, epidemiologic and anatomical factors [2,3,4,5]. The combination of gemcitabine and cisplatin chemotherapy (GemCis) has been considered as the standard chemotherapy regimen since the pivotal ABC-02 trial showed superior efficacy and survival outcomes of GemCis over gemcitabine monotherapy [6]. Nevertheless, the clinical outcome of advanced BTC remains unsatisfactory, with the overall response rate to GemCis chemotherapy at around 13–26%, and the 5-year survival rates less than 10% [6,7,8]. 

Human epidermal growth factor receptor 2 (HER2) is known for its role in tumourigenesis in many cancers [9,10]. In breast and stomach cancer, it serves as a prognostic and predictive biomarker [11,12], and, therefore, HER2-targeted therapy has been adopted as a major part of the current standard regimen in HER2 overexpressed or amplified breast and stomach cancer [13,14,15]. Trastuzumab, the first recombinant humanised monoclonal antibody against HER2, has been widely used as one of the leading HER2-targeted agents. Recently, various trastuzumab biosimilars such as Herzuma (trastuzumab-pkrb) [16,17], Ogivri (trastuzumab-dkst) [18], Ontruzant (trastuzumab-dttb) [19], Trazimera (trastuzumab-qyyp) [20] and Kanjinti (trastuzumab-anns) [21] have been developed and approved. As these agents show efficacy and safety similar to trastuzumab, their use has become increasingly popular.

With the recent understanding of the molecular landscape of BTC, potential therapeutic targets, including HER2, are being explored for personalised treatment. In BTC, the incidence of HER2 overexpression is around 10–16% in gallbladder cancer, 5–9% in extrahepatic cholangiocarcinoma and 1% in intrahepatic cholangiocarcinoma, although reports vary [22,23,24]. The role of HER2-targeted therapy in BTC is unknown, with a small number of anecdotal reports present [25,26,27]. Given the poor prognosis on the current standard regimen and the considerable rates of HER2-positive cancers, therapeutic implications of HER2 in BTC need further investigation, although prospective evidence on this subject is lacking. 

Therefore, we conducted a pilot study to investigate the feasibility of HER2-targeted therapy and the preliminary efficacy and safety of first-line trastuzumab-pkrb plus GemCis in patients with unresectable or metastatic BTC.

## 2. Results

**Figure 1:**
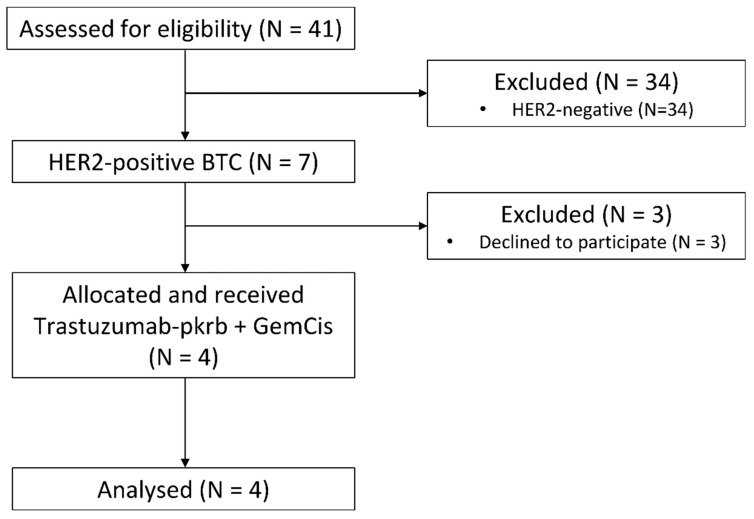
CONSORT diagram. Abbreviations: HER2, human epidermal growth factor receptor 2; BTC, biliary tract cancer; GemCis, gemcitabine and cisplatin.

From August 2019 to August 2020, 41 patients were screened for eligibility at the Asan Medical Center, Seoul, Korea. Seven HER2-positive patients were identified (17.1%), and four patients proceeded to receive trastuzumab-pkrb plus GemCis (Figure 1).

### 2.1. Patient Characteristics

The baseline characteristics of the study participants are shown in Table 1. The median age was 72.5 years (range: 62–75), and one patient was male (25.0%). The primary tumour location was gallbladder in one patient, intrahepatic bile duct in one patient and extrahepatic bile duct in two patients. A total of 33 patients who were screened but not eligible for this study received GemCis (N = 25, 75.8%), followed by GemCis plus nab-paclitaxel (N = 5, 15.2%) or GemCis plus pembrolizumab (N = 2, 6.1%).

Table 2 shows the baseline characteristics of the screened patients by HER2 positivity. Patients who had HER2-positive tumours (N = 7) tended to be older, with a median age of 69 years, compared with the median age of 59.5 years in the HER2-negative group (N = 34). The most common primary tumour location was the gallbladder (N = 3, 42.9%) and extrahepatic bile duct (N = 3, 42.9%) in the HER2-positive group, while gallbladder (N = 14, 41.2%) and intrahepatic bile duct cancer (N = 12, 35.3%) were predominant in the HER2-negative group.

### 2.2. Efficacy

**Figure 2:**
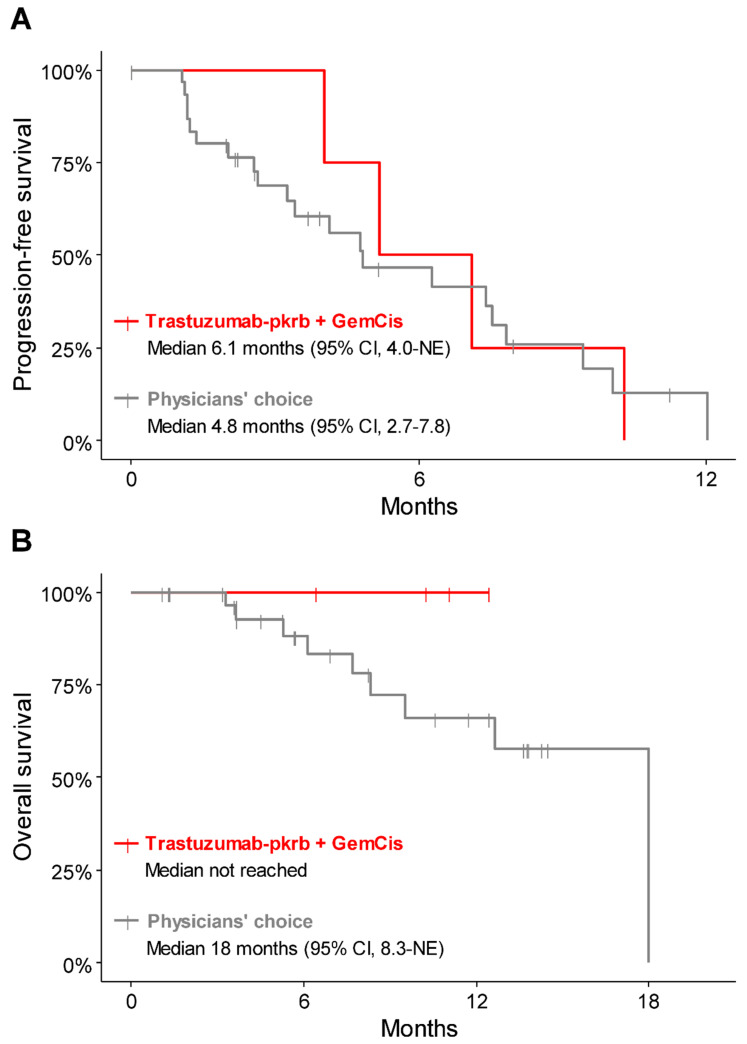
Kaplan–Meier survival curve for patients treated with trastuzumab-pkrb + GemCis and physicians’ choice of chemotherapy. (**A**) progression-free survival, (**B**) overall survival. Abbreviations: GemCis, gemcitabine and cisplatin.

During the median follow-up of 10.6 months (95% confidence interval (CI), 6.41—not estimated (NE)), all four patients receiving trastuzumab-pkrb plus GemCis discontinued treatment owing to disease progression, but were alive at the time of data cutoff. The best overall response was a partial response (PR) in two patients (50.0%) and stable disease (SD) in two patients (50.0%), resulting in an overall response rate (ORR) of 50.0% and a disease-control rate (DCR) of 100% (Table 3, Supplementary Figure S1). The median progression-free survival (PFS) was 6.1 months (95% CI, 4.0—NE), and the median overall survival (OS) was not reached (Figure 2).

For reference, the clinical outcome of the 33 patients who were screened but did not enter the clinical study and received the physician’s choice of chemotherapy were also analysed. The median follow-up duration of these patients was 10.5 months (95% CI, 5.3–13.7), the ORR was 17.2% with all partial responses and the DCR was 69.0% (Table 3). The median PFS was 4.8 months (95% CI, 2.7–7.8), and the median OS was 18 months (95% CI, 8.3—NE). The PFS and OS of three patients with HER2-positive disease but treated with physicians’ choice of chemotherapy were 1.95 months (95% CI, 1.25—NE) and 5.29 months (95% CI, NE), respectively (Supplementary Figure S2).

### 2.3. Second-Line Chemotherapy

During follow-up, all four patients treated with trastuzumab-pkrb plus GemCis experienced disease progression and proceeded to second-line chemotherapy. Of the 33 screened patients who were treated with physicians’ choice of chemotherapy, 21 patients (63.6%) experienced disease progression and 18 (85.7%) proceeded to second-line chemotherapy. Overall, the most commonly used second-line regimen was capecitabine plus cisplatin (N = 9, 40.9%), followed by pembrolizumab monotherapy (N = 6, 27.3%).

### 2.4. Safety

During the trastuzumab-pkrb plus GemCis treatment, all four patients experienced grade 3 toxicities, but no grade 4 toxicity was reported. The most common grade 3 toxicity was neutropenia (N = 3, 75.0%), but febrile neutropenia did not occur. Other common grade 3 toxicity effects were anaemia (N = 1, 25.0%), nausea (N = 1, 25.0%) and increased serum bilirubin (N = 1, 25.0%) (Table 4). No patient demonstrated ≥10% decrease in the left ventricular ejection fraction from baseline on repeated multigated cardiac blood pool (MUGA) scans. No patient discontinued treatment due to adverse events.

### 2.5. Mutational Profile of HER2-Positive Tumours

Six out of seven HER2-positive BTC patients screened for this study had targeted next-generation sequencing results. *HER2* amplification by next-generation sequencing, defined as an estimated copy number of 5 or more, was detected in four out of six patients. The most commonly mutated gene was *TP53* (N = 5, 83.3%), followed by *FGFR4, U2AF1, ROS1, NF1* and *MEN2* (N = 2, 33.3% for each). *KRAS* and *BRCA1* mutations were noted in one patient each. No patient had *IDH1/2* mutation, *MET* amplification, *FGFR2* fusion or *BRAF* mutation. Other than *HER2* amplification, the most common copy number alteration was *CDK12* amplification (N = 3, 50.0%), followed by *RARA* amplification and *CDKN2A/B* loss (N = 2, 33.3% for each). Mutations in DNA damage repair genes were found in four patients (66.7%). All patients had microsatellite-stable tumours. 

## 3. Discussion

The results of this pilot study showed that HER2 is a feasible target for the management of patients with advanced BTC, and the trastuzumab-pkrb plus GemCis combination has encouraging preliminary efficacy in HER2-positive, previously untreated, advanced BTC patients without new safety issues. To the best of the authors’ knowledge, this is the first prospective study that evaluated the feasibility of HER2-targeted therapy in advanced BTC. 

In this study, trastuzumab-pkrb plus GemCis showed a DCR and ORR of 100% and 50.0%, respectively, a median PFS of 6.1 months and a 6 month survival rate of 100%. Although this study is not designed for direct comparison, the survival outcome tended to be more favourable for the trastuzumab-pkrb plus GemCis treated patients than for the patients not enrolled for this study and treated with physicians’ choice of chemotherapy. It is also worth mentioning that the ORR and DCR with trastuzumab-pkrb plus GemCis in this study were likely to be higher than those with GemCis in the pivotal ABC-02 trial (26% and 81%, respectively) and our previous retrospective analysis (13% and 66%, respectively) [6,8]. Although PFS seemed to be shorter than the results of the ABC-02 trial (median 8.0 months), our data were likely to be better than the results (median 5.2–5.8 months) of our previous large-scale retrospective data and a Japanese phase 2 study for GemCis [8,28]. High ORR with trastuzumab-pkrb plus GemCis in our study was in line with the results of earlier small-sized studies showing tumour response to HER2-targeted agents in the majority of included BTC patients [25,26,27,29,30]. Trastuzumab-pkrb plus GemCis was generally well-tolerated, and no patient discontinued treatment due to adverse events. There was no new safety signal identified with trastuzumab-pkrb. 

The prevalence of HER2-positive BTC in this study was 17%. We defined HER2 positivity by HER2 overexpression, according to the criteria for gastric cancer [15,31,32]. The incidence of HER2 overexpressing disease in this study is consistent with previous reports showing 18%, 8% and 27% of patients with gallbladder cancer, intrahepatic cholangiocarcinoma and extrahepatic cholangiocarcinoma, respectively. Previous studies used heterogeneous definitions of HER2 positivity in BTC [24,25,27,30]. In BTC, a fair correlation of 79% between HER2 overexpression by immunohistochemistry (IHC) and amplification by in situ hybridisation has been reported. In contrast, a previous meta-analysis suggested low correlation between these two tests in the subgroup of patients with HER2 IHC+ tumours [24]. Intratumoral HER2 heterogeneity is also common in BTC as it is in other solid cancers, including gastric cancer [33]. Not surprisingly, we noted cases with discrepant HER2 status by IHC/silver in situ hybridisation (SISH) and targeted sequencing in our study. For example, 2 out of 16 cases classified as HER2-negative tumours at study screening were found to have *HER2* amplification at subsequent targeted sequencing from the same tissue. In gastric or colorectal cancers, a high correlation in HER2 positivity assessed by IHC/in situ hybridisation and by targeted sequencing was reported [34,35]. However, as various factors such as tumour heterogeneity or sample quality might cause a discrepancy between different diagnostic methods, the optimal definition of HER2-positive tumours in BTC needs to be further delineated. Nonetheless, data suggest that a considerable proportion of BTCs, especially gallbladder or extrahepatic cholangiocarcinomas, have positive HER2 results, and HER2 testing should have a higher value in these patients [22,24,36].

Current findings suggest that HER2 is a feasible target for improving efficacy outcomes in unresectable or metastatic BTC, considering that HER-2 targeted therapy has dramatically improved clinical outcomes in patients with HER2-positive breast and gastric cancers. Future prospective randomised trials using currently available or investigational HER2 targeted therapy such as trastuzumab, pertuzumab, T-DM1 or trastuzumab deruxtecan are warranted. 

This study was limited by the small sample size and the lack of long-term follow-up data. Additionally, the survival outcome of physicians’ choice of chemotherapy treated patients presented in this study should be interpreted in the context of a reference, not for direct comparison. However, the follow-up duration of this study (10.6 months) was comparable to that of the pivotal ABC-02 trial (8.2 months), and all patients were followed up until disease progression. Another strength of our study is that we assessed HER2 status by uniform predefined criteria, and reported the considerable incidence of HER2-positive tumours in a prospectively recruited patient population. Being the first prospective study assessing the efficacy and safety of HER2-targeted treatment in combination with current standard therapy for the first-line treatment of advanced BTC, the authors believe that this study provides valuable preliminary results on the feasibility of HER2-targeted treatment in this patient population despite its small sample size. 

## 4. Materials and Methods

### 4.1. Study Design

This study is an open-label, single-arm, single-centre, investigator-initiated trial. The study protocol was approved by the Institutional Review Board, Asan Medical Center. This study was conducted in accordance with the Declaration of Helsinki and the International Conference on Harmonization guidelines for Good Clinical Practice. All participants provided written informed consent (ClinicalTrials.gov identifier: NCT03613168).

### 4.2. Eligibility

Patients with locally advanced, initially metastatic or recurrent BTC were included in this study if they met the following eligibility criteria: histologically confirmed adenocarcinoma of the bile duct, HER2 overexpression, age ≥19 years, Eastern Cooperative Oncology Group (ECOG) performance status of 0–1, at least one measurable lesion per the Response Evaluation Criteria in Solid Tumours (RECIST) version 1.1, at least 3 months for life expectancy and no prior chemotherapy in a palliative setting. Patients were excluded if they had insufficient cardiac function, defined as baseline left ventricular ejection fraction < 50%, assessed by either echocardiography or a multigated cardiac blood pool scan (MUGA scan); known history of heart disease including congestive heart failure or significant valvular heart disease; angina that requires medication; uncontrolled hypertension/fatal arrhythmia or evidence of transmural myocardial infarction on an electrocardiogram. Potentially eligible patients were screened for HER2 overexpression by an independent pathologist (YSP). HER2 positivity was defined either as HER2 immunohistochemistry (IHC) 2+ and positive for silver in situ hybridisation (SISH+), or HER2 IHC 3+ (Supplementary Table S3) [15,31,32,37]. Additional targeted next-generation sequencing, using an in-house panel of the Asan Medical Center, Seoul, Republic of Korea (OncoPanel AMC, versions 4), was performed at the discretion of the physician. 

### 4.3. Treatment and Endpoints

Patients who met all eligibility criteria received trastuzumab-pkrb (Herzuma^®^, 8 mg/kg at day 1 of the first cycle and 6 mg/kg at day 1 of subsequent cycles), gemcitabine (1000 mg/m^2^ at day 1 and day 8) and cisplatin (25 mg/m^2^ at day 1 and day 8) every 3 weeks. MUGA scan or echocardiography was performed every four cycles. Patients who were screened but not eligible received the treatment at the discretion of the attending physician. Trastuzumab-pkrb was generously provided by Celltrion.

Primary endpoints were the best overall response according to the RECIST version 1.1 and adverse events were graded by the National Cancer Institute Common Terminology Criteria for Adverse Events (NCI-CTCAE) version 4.03. Secondary endpoints were progression-free survival (PFS), defined by the time between the initiation of chemotherapy and disease progression or death, whichever occurred first, and overall survival (OS), defined by the time between the initiation of chemotherapy and the date of death of any cause. 

### 4.4. Statistical Analyses 

Patient characteristics and toxicities were assessed using a descriptive method. Survival outcomes were estimated using the Kaplan–Meier method and compared by a log-rank test. All tests were two-sided, and a *p*-value of <0.05 was considered statistically significant. Statistical analyses were performed using R version 4.0.1 (R Foundation for Statistical Computing, Vienna, Austria).

## 5. Conclusions

Trastuzumab-pkrb in combination with GemCis chemotherapy showed promising preliminary efficacy and tolerability in patients with HER2-positive advanced BTC. 

## Figures and Tables

**Table 1:** Baseline characteristics.

Characteristics	Trastuzumab-pkrb + GemCis N = 4	Screened But Not Entered To Clinical Study N = 37
**Age**		
Median (range)	72.5 (62–75)	60 (39–75)
**Sex**		
Male	1 (25.0%)	17 (45.9%)
**ECOG PS**		
0	1 (25.0%)	5 (13.5%)
1	3 (75.0%)	32 (86.5%)
**Primary tumour location**		
Gallbladder	1 (25.0%)	16 (43.2%)
Intrahepatic	1 (25.0%)	12 (32.4%)
Extrahepatic	2 (50.0%)	9 (24.3%)
**Aetiology**		
Hepatitis B	0 (0.0%)	2 (5.4%)
Hepatitis C	0 (0.0%)	1 (2.7%)
Clonorchis infection	1 (25.0%)	0 (0.0%)
APBDU/Choledochal cyst	0 (0.0%)	2 (5.4%)
Unknown	3 (75.0%)	32 (86.5%)
**Histologic grade**		
Well differentiated	0 (0.0%)	4 (10.8%)
Moderately differentiated	2 (50.0%)	21 (56.8%)
Poorly differentiated	2 (50.0%)	9 (24.3%)
Not specified	0 (0.0%)	3 (8.1%)
**Disease status at palliative first-line chemotherapy**		
Initially metastatic	3 (75.0%)	20 (54.1%)
Locally advanced	0 (0.0%)	2 (5.4%)
Recurrent after curative surgery	1 (25.0%)	15 (40.5%)
**No of metastatic sites**		
0	1 (25.0%)	5 (13.9%)
1	1 (25.0%)	16 (43.2%)
≥2	2 (50.0%)	16 (43.2%)
**Sites of metastasis**		
Liver	1 (25.0%)	19 (51.4%)
Distant lymph node	2 (50.0%)	21 (56.8%)
Peritoneum	1 (25.0%)	12 (32.4%)
Lung	1 (25.0%)	5 (13.5%)
**HER2 status**		
Positive, IHC 3+	0 (0.0%)	2 (5.4%)
Positive, IHC 2+ SISH+	4 (100.0%)	1 (2.7%)
Negative, IHC 2+ SISH-	0 (0.0%)	4 (10.8%)
Negative, IHC 0-1+	0 (0.0%)	30 (81.1%)
**Level of CA 19-9 at palliative first-line chemotherapy**		
Normal	0 (0.0%)	12 (32.4%)
Elevated	4 (100.0%)	23 (62.2%)
Not assessed	0 (0.0%)	2 (5.4%)
**Palliative first-line chemotherapy regimen**	N = 4	N = 33
Trastuzumab-pkrb + GemCis	4 (100.0%)	0 (0.0%)
GemCis	0 (0.0%)	25 (75.8%)
GemCis + nab-paclitaxel	0 (0.0%)	5 (15.2%)
GemCis + pembrolizumab	0 (0.0%)	2 (6.1%)
Capecitabine + cisplatin	0 (0.0%)	1 (3.0%)

Abbreviations: GemCis, gemcitabine and cisplatin; ECOG PS, Eastern Cooperative Oncology Group performance status; APBDU, anomalous pancreaticobiliary ductal union; SISH; silver in situ hybridisation; IHC, immunohistochemistry; CA19-9; carbohydrate antigen 19-9.

**Table 2:** Baseline characteristics by HER2 positivity.

Characteristics	HER2-Positive N = 7	HER2-Negative N = 34	*p* Value
**Age**			0.031
Median (range)	69 (60–75)	59.5 (39–75)	
**Sex**			0.188
Male	1 (14.3%)	17 (50.0%)	
**ECOG PS at diagnosis**			1.000
0	1 (14.3%)	5 (14.7%)	
1	6 (85.7%)	29 (85.3%)	
**Primary tumour location**			0.445
Gallbladder	3 (42.9%)	14 (41.2%)	
Intrahepatic	1 (14.3%)	12 (35.3%)	
Extrahepatic	3 (42.9%)	8 (23.5%)	
**Aetiology**			0.208
Hepatitis B	0 (0.0%)	2 (5.9%)	
Hepatitis C	0 (0.0%)	1 (2.9%)	
Clonorchis infection	1 (14.3%)	0 (0.0%)	
APBDU/Choledochal cyst	0 (0.0%)	2 (5.9%)	
Unknown	6 (85.7%)	29 (85.3%)	
**Histologic grade**			0.614
Well differentiated	0 (0.0%)	4 (11.8%)	
Moderately differentiated	5 (71.4%)	18 (52.9%)	
Poorly differentiated	2 (28.6%)	9 (26.5%)	
Not specified	0 (0.0%)	3 (8.8%)	
**Disease status at palliative first-line chemotherapy**			0.606
Initially metastatic	5 (71.4%)	18 (52.9%)	
Locally advanced	0 (0.0%)	2 (5.9%)	
Recurrent after curative surgery	2 (28.6%)	14 (41.2%)	
**No of metastatic sites**			0.756
0	1 (14.3%)	5 (15.2%)	
1	2 (28.6%)	15 (44.1%)	
≥2	4 (57.1%)	14 (42.4%)	
**Sites of metastasis**			
Liver	3 (42.9%)	17 (50.0%)	1
Distant lymph node	4 (57.1%)	19 (55.9%)	1
Peritoneum	1 (14.3%)	12 (35.3%)	0.521
Lung	3 (42.9%)	3 (8.8%)	0.083
**HER2 status**			< 0.001
Positive, IHC 3+	2 (28.6%)	0 (0.0%)	
Positive, IHC 2+ SISH+	5 (71.4%)	0 (0.0%)	
Negative, IHC 2+ SISH-	0 (0.0%)	4 (11.8%)	
Negative, IHC 0-1+	0 (0.0%)	30 (88.2%)	
**Level of CA19-9 at palliative first-line chemotherapy**			0.135
Normal	0 (0.0%)	12 (35.3%)	
Elevated	7 (100.0%)	20 (58.8%)	
Not assessed	0 (0.0%)	2 (5.9%)	

Abbreviations: GemCis, gemcitabine and cisplatin; ECOG PS, Eastern Cooperative Oncology Group performance status; APBDU, anomalous pancreaticobiliary ductal union; SISH; silver in situ hybridisation; IHC, immunohistochemistry; CA19-9; carbohydrate antigen 19-9.

**Table 3:** Tumour response to treatment.

Tumour Response	Trastuzumab-pkrb + GemCis	Physicians’ Choice
	Patients with measurable lesion	
**Best response**	**N = 4**	**N = 29**
Complete response	0	0
Partial response	2 (50.0%)	5 (17.2%)
Stable disease	2 (50.0%)	15 (51.7%)
Progressive disease	0 (0.0%)	6 (20.7%)
Not evaluable	0 (0.0%)	3 (10.3%)
**Overall response rate**	2 (50.0%)	5 (17.2%)
**Disease control rate**	4 (100.0%)	20 (69.0%)

Abbreviations: GemCis, gemcitabine and cisplatin.

**Table 4:** Adverse events of trastuzumab-pkrb + GemCis.

Adverse Events	Any Grade	Grade 3
Any adverse events	4 (100%)	4 (100%)
Haematologic		
Leukopenia	4 (100%)	0 (0.0%)
Neutropenia	4 (100%)	3 (75.0%)
Anaemia	4 (100%)	1 (25.0%)
Thrombocytopenia	1 (25.0%)	0 (0.0%)
Febrile neutropenia	0 (0.0%)	0 (0.0%)
Non-haematologic		
Anorexia	2 (50.0%)	0 (0.0%)
Nausea	2 (50.0%)	1 (25.0%)
Fatigue	1 (25.0%)	0 (0.0%)
Fever	1 (25.0%)	0 (0.0%)
Tremor	1 (25.0%)	1 (25.0%)
Allergic reaction	2 (50.0%)	0 (0.0%)
Hypoalbuminemia	2 (50.0%)	0 (0.0%)
Increased AST	1 (25.0%)	0 (0.0%)
Increased ALT	1 (25.0%)	0 (0.0%)
Increased ALP	2 (50.0%)	0 (0.0%)
Increased bilirubin	1 (25.0%)	1 (25.0%)

Abbreviations: AST, aspartate aminotransferase; ALT, alanine aminotransferase; ALP, alkaline phosphatase.

## Data Availability

The data presented in this study are available on request from the corresponding author.

## Supplementary Materials

The following are available online at https://www.mdpi.com/2072-6694/13/2/161/s1, Figure S1: Waterfall plot of the best percent changes in the sum of the target lesions, according to the (A) HER2 amplification status by targeted sequencing, and (B) primary tumour location. Figure S2: (A) progression-free survival and (B) overall survival of patients according to the HER2 status and treatment. Table S3: HER2 immunohistochemistry.
