# Supplementary material for: Feasibility of HER2-Targeted Therapy in Advanced Biliary Tract Cancer: A Prospective Pilot Study of Trastuzumab Biosimilar in Combination with Gemcitabine Plus Cisplatin
Source: Cancers (Basel). 2021 Jan 6;13(2):161. doi: 10.3390/cancers13020161 (PMC7825072; doi:10.3390/cancers13020161)
Supplement: Supplementary file 1 [file cancers-13-00161-s001.pdf]

Supplementary figure S1. Waterfall plot of the best percent changes in the sum of the target lesions, according to the (A) HER2 amplification status by targeted sequencing, and (B) primary tumour location.

A

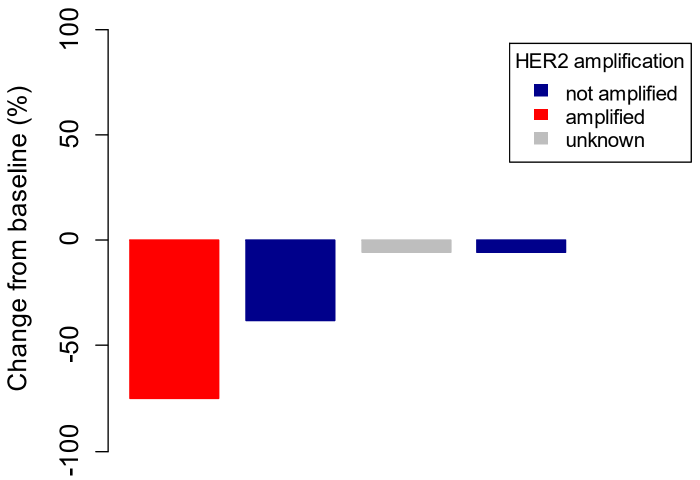

B

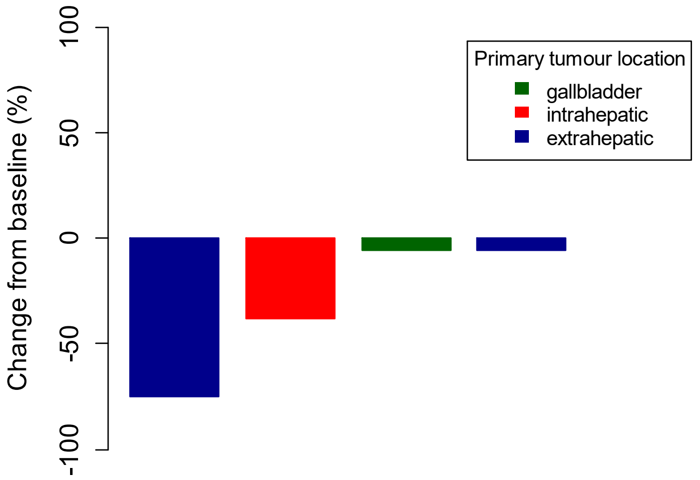

Supplementary Fig. S2 (A) progression-free survival and (B) overall survival of patients according to the HER2 status and treatment.

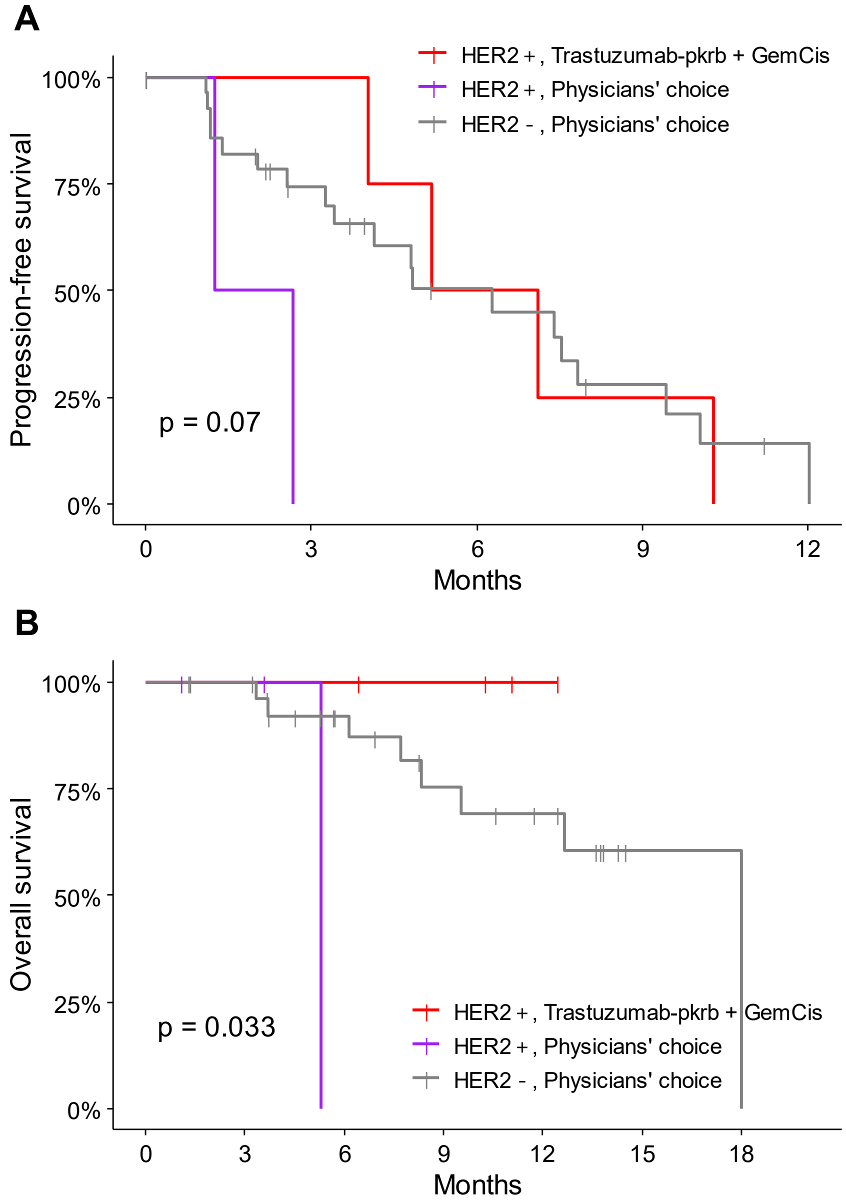

Abbreviations: HER2, human epidermal growth factor receptor 2, GemCis; gemcitabine and cisplatin.

Supplementary Table S3. HER2 immunochemistry†

	**Surgical specimen staining pattern**	**Biopsy specimen staining pattern**	**HER2 overexpression assessment**
**0**	No reactivity or membranous reactivity in <10% of tumour cells	No reactivity or no membranous reactivity in any tumour cell	Negative
**1+**	Faint or barely perceptible membranous reactivity in ≥10% of tumour cells; cells are reactive only in part of their membrane	Tumour cell cluster with a faint or barely perceptible membranous reactivity irrespective of percentage of tumour cells stained	Negative
**2+**	Weak to moderate complete, basolateral or lateral membranous reactivity in ≥10% of tumour cells	Tumour cell cluster with a weak to moderate complete, basolateral or lateral membranous reactivity irrespective of percentage of tumour cells stained	Equivocal
**3+**	Strong complete, basolateral or lateral membranous reactivity in ≥10% of tumour cells	Tumour cell cluster with a strong complete, basolateral or lateral membranous reactivity irrespective of percentage of tumour cells stained	Positive

†HER2 positivity was defined either as HER2 immunohistochemistry (IHC) 2+ and positive for silver in situ hybridisation (SISH+), or HER2 IHC 3+. SISH was interpreted as per the ASCO/CAP 2018 guideline.
